# Investigation of Ionization Potential in Quantum Dots Using the Stratified Stochastic Enumeration of Molecular Orbitals Method

**DOI:** 10.1021/acs.jctc.2c00329

**Published:** 2022-09-22

**Authors:** Nicole Spanedda, Peter F. McLaughlin, Jessica J. Beyer, Arindam Chakraborty

**Affiliations:** † Department of Chemistry, Syracuse University, Syracuse, New York 13244, United States; ‡ Keck Science Department, Scripps College, Claremont, California 91711, United States

## Abstract

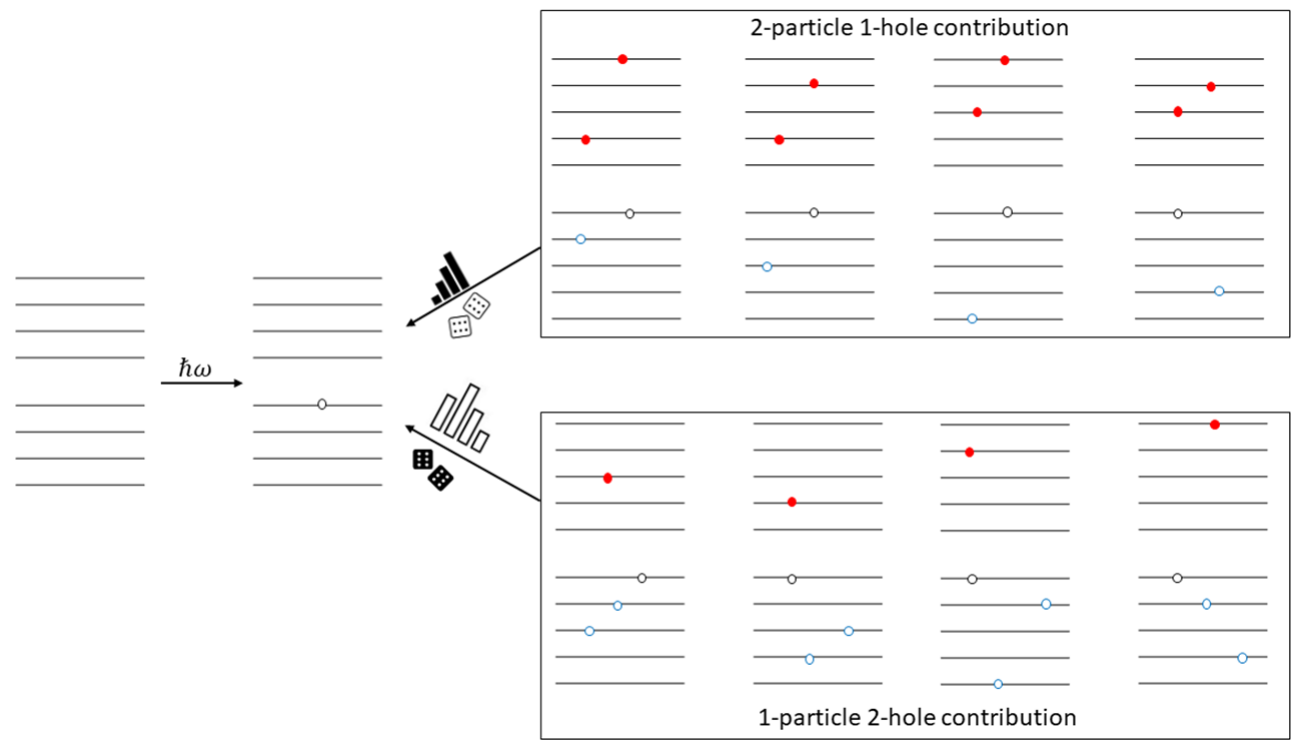

The overarching goal of this work is to investigate the size-dependent characteristics of the ionization potential of PbS and CdS quantum dots. The ionization potentials of quantum dots provide critical information about the energies of occupied states, which can then be used to quantify the electron-removal characteristics of quantum dots. The energy of the highest-occupied molecular orbital is used to understand electron-transfer processes when invesigating the energy-level alignment between quantum dots and electron-accepting ligands. Ionization potential is also important for investigating and interpreting electron-detachment processes induced by light (photoelectron spectra), external voltage (chemiresistance), and collision with other electrons (impact ionization). Accurate first-principles calculations of ionization potential continue to be challenging because of the computational cost associated with the construction of the frequency-dependent self-energy operator and the numerical solution of the associated Dyson equation. The computational cost becomes prohibitive as the system size increases because of the large number of 2particle-1hole (2p1h) and 1particle-2hole (1p2h) terms needed for the calculation. In this work, we present the Stratified Stochastic Enumeration of Molecular Orbitals (SSE-MO) method for efficient construction of the self-energy operator. The SSE-MO method is a real-space method and the central strategy of this method is to use stochastically enumerated sampling of molecular orbitals and molecular-orbital indices for the construction of the 2p1h and 1p2h terms. This is achieved by first constructing a composite MO-index Cartesian coordinate space followed by transformation of the frequency-dependent self-energy operator to this composite space. The evaluation of both the real and imaginary components of the self-energy operator is performed using a stratified Monte Carlo technique. The SSE-MO method was used to calculate the ionization potentials and the frequency-dependent spectral functions for a series of PbS and CdS quantum dots by solving the Dyson equation using both single-shot and iterative procedures. The ionization potentials for both PbS and CdS quantum dots were found to decrease with increasing dot size. Analysis of the frequency-dependent spectral functions revealed that for PbS quantum dots the intermediate dot size exhibited a longer relative lifetime whereas in CdS the smallest dot size had the longest relative lifetime. The results from these calculations demonstrate the efficacy of the SSE-MO method for calculating accurate ionization potentials and spectral functions of chemical systems.

## 1 Introduction

Ionization potential (IP) ω (or ionization energy) is defined as the energy needed to remove an electron from a chemical system.
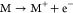
1
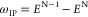
2The ejection of the electron can be facilitated using incident photons, scattering by high-energy electrons, or applying a strong electric field. As one of the fundamental properties of a material, IP has relevance to the areas of photovoltaics, mass spectrometry, photoelectron spectroscopy, electrochemistry, photocatalysis, and light-induced electron-transfer processes. The IP of atoms, molecules, and various chemical compounds is a quantity of interest when performing X-ray spectroscopy. Recently, it has been found that small gas-phase polyatomic molecules with a heavy atom, such as iodomethane, when bombarded with hard X-ray pulses display surprisingly enhanced ionization relative to an individual heavy atom with the same absorption cross-section.^1^ Following the excitation of an electron from an inner orbital of one atom, another electron from a higher energy orbital of the same atom can occupy this inner orbital. Instead of undergoing de-excitation, the newly excited electron can transfer energy via photon emission by ionizing an electron from an outer orbital of a neighboring atom. This process is called interatomic Coulombic decay (ICD).^2,3^ The IPs of both the inner and outer orbitals of molecules, dimers, and clusters influence which de-excitation mechanism occurs in a highly excited neutral or a highly excited ionized state of these types of systems. Knowledge of the IPs of these systems’ inner and outer orbitals can assist in the prediction of which de-excitation mechanism is likely to occur in a system of interest.^4−9^ High precision IP measurements in atoms and molecules provide important information about electron–electron correlation and serves as a benchmark for the development and testing of theories. Ionization potential also is a quantity of interest in biological systems. For example, ionizing radiation causes permanent heritable DNA damage,^10^ and the IPs of nucleic acid tautomers are quantities of interest, due to the role of these molecules in cancer.^11^

In quantum dots (QDs) and nanomaterials, knowledge of the IP for ionization from HOMO serves as an important metric for quantifying electron-transfer rates.^12−18^ Photoejection of electrons by X-ray and UV radiation has been used to study valence-band states in QDs.^19^ Cyclic voltametry has also been used to calculate HOMO energies in QDs and IPs.^20^ Transient photoemission in two-photon experiments has provided information on the energy levels of unoccupied orbitals.^21^ Knowledge of the relative positions of the HOMO and lowest unoccupied molecular orbital (LUMO) levels of a QD with respect to the surface ligands is an important factor in extraction of a hot carrier from the QD.^19^

In molecular quantum chemistry, the simplest approximation of IP (denoted as ω_*i*_^0^) is given by the Koopmans’ approximation, where the exact IP is approximated as the negative of the orbital energies.
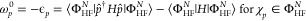
3Koopmans’ treatment utilizes the Hartree–Fock (HF) approximation to obtain a single N-electron Slater determinant from which an electron from one of the occupied states is annihilated, as shown in eq 3. Although Koopmans’ theorem is limited by the use of single Slater determinants and does not account for orbital relaxation and electron–electron correlation effects, it still provides an acceptable first approximation for the IP of a system of interest.^22^ Going beyond Koopmans’ approximation by including the effect of electron−electron correlation can be achieved in a variety of different ways such as with electron-propagator methods,^23−31^ algebraic diagrammatic construction (ADC),^32,33^ equation-of-motion coupled-cluster (IP-EOM-CCSD),^34−39^ many-body perturbation theory (MBPT),^40,41^ GW method,^42^ correlated-orbital theory (COT),^43^ and time-dependent density functional theory (TDDFT).^44−46^ The IP of the HOMO has a special significance in DFT because of Janak’s theorem and plays a prominent role in the development and testing of DFT functionals.^47^ Without loss of generality, the many-body correction to the IP can always be written as,
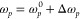
4where Δ*ω*_*p*_ accounts for all of the correction terms missing from the Koopmans’ approximation; post-HF methods mentioned earlier offer different approximations and formulations for calculating Δ*ω*_*p*_. However, efficient first-principles calculation of Δ*ω*_*p*_ for large chemical systems continues to be challenging and is an active field of research. Using the electron-propagator method, Ortiz and co-workers developed a series of approximations that offer an order-by-order treatment of electron correlation to the many-body correction for IPs.^23−25,48,49^ Open-shell systems possess additional complexities compared to their closed-shell counterparts, and the spin-flip EOM-IP approach has been used to treat open-shell systems.^50^ In the GW formulation, the projective eigen decomposition of the dielectric screening (PDEP) algorithm has been used for calculating the quasiparticle gap of QDs.^42^ In recent works, methods using stochastic techniques have been demonstrated to achieve the low-scaling needed for applications to large chemical systems. Specifically, the use of stochastic orbitals in the stochastic Green’s function method developed by R. Baer can be used for the calculation of IPs.^51^ A different strategy of combining the Laplace-transformed expression of the self-energy operator with a real-space Monte Carlo integration scheme developed by Hirata and co-workers has been used for the calculation of IPs at the second-order MP2 level.^52^ The approach was recently extended for ground-state MP4 level.^53^ In a related work by Li et al., the Laplace-transformed MP2 approach has been combined with the density-of-states approach to reduce the overall computational cost of the MP2 calculation.^54^ This approach demonstrated the effectiveness of using the intrinsic degeneracies present in chemical systems to reduce the overall cost of MP2 calculations.

**Figure 1:**
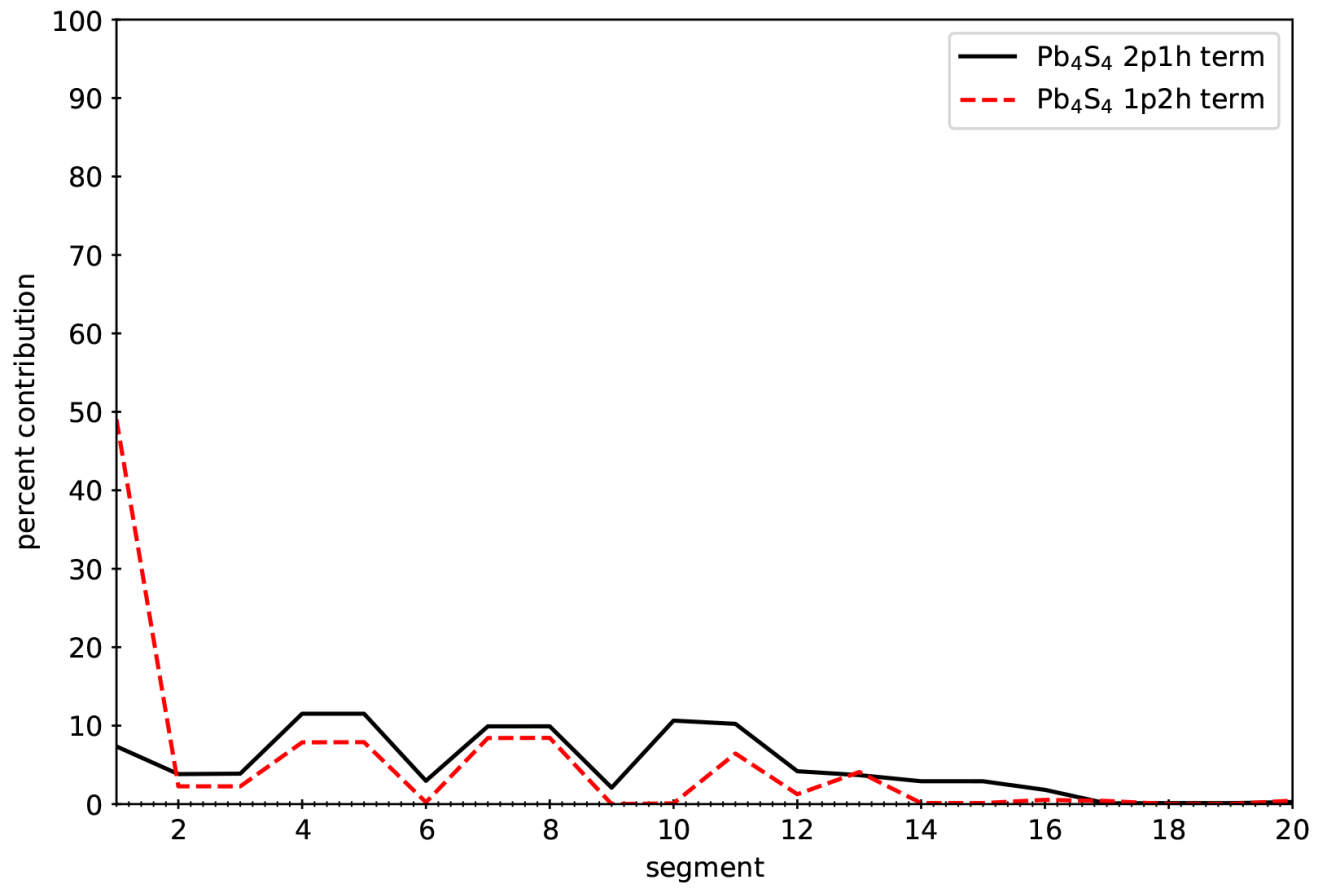
Percent contribution of terms *A*_*i*_^2p1h^ and *A*_*i*_^1p2h^ (defined in eq 5) to the total self-energy as a function of hole index (*i*) for Pb_4_S_4_.

In this article, we present the stratified stochastic enumeration of molecular orbitals (SSE-MO) method for the efficient computation of the IP through the iterative solution of the Dyson equation. The SSE-MO method was originally inspired by the 2013 paper, “*Stochastic Enumeration Method for Counting NP-Hard Problems*” by Rubinstein.^55^ The original stochastic enumeration by Rubinstein was based on the importance sampling scheme. In the field of computer science, the stochastic enumeration technique has been applied to traversing deep tree structures and implementing backtracking algorithms.^55^ For the SSE-MO method, we have combined stochastic enumeration with stratified sampling to perform the necessary summations over a direct-product space of molecular orbital indices and 6D Cartesian coordinate space. The reduced computational cost of the SSE-MO method allowed us to investigate the full frequency-dependent pole structure of the 1-particle Green’s function by an iterative solution of the Dyson equation. In this work, the SSE-MO method was used to investigate IPs of PbS and CdS QDs. The motivation for the SSE-MO method comes from the fact that not all terms contribute equally to the overall self-energy operator. A similar observation has also been made for the density-of-state MP2 method.^54^ For example, for the Pb_4_S_4_ system, the contribution for each term as a function of the hole-index, (*i*), is presented in Figure 1. Without loss of generality, the self-energy operator can be written as the sum of the 2-particle 1-hole (2p1h) and 1-particle 2-hole (1p2h) terms as shown in eq 5.
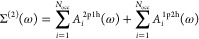
5It is seen that some terms contribute more than other terms. The SSE-MO method aims to distribute the computation effort used to calculate of the overall self-energy operator in proportion to the contribution of each of the terms and their associated errors. Here, we provide a general formulation of stratified stochastic tensor contraction (section 2.1), details about the construction of the self-energy operator (section 2.2), formulation of stochastic stratified sampling in MO space (section 2.3), performing low-discrepancy sampling (section 2.5), construction of the control-variate functions for the real-space integrals (section 2.6), performing joint MO-space real-space stratified sampling (section 2.7), the iterative solution of the Dyson equation (section 2.2), and the calculation of pole-strengths of the Green’s function and calculation of higher-order derivatives of the self-energy operator (section 2.9). Benchmark calculations on well-studied systems and new results on PbS and CdS QDs are presented in section 3. Comparisons to existing methods and future directions are discussed in section 4.

## 2 Theory

### 2.1 Stratified Stochastic Enumerated Tensor Contraction

As an introduction to the application of SSE for the calculation of the self-energy operator, we present the SSE approach for performing a general N-index tensor contraction. We start by considering the following general tensor contraction *S* = Tr{**ABCD**}.
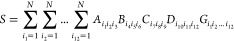
6One situation for which this type of tensor contraction is encountered is when the integration of a 4-point kernel in real space ⟨*A*(**r**_1_)*B*(**r**_2_)*C*(**r**_3_)*D*(**r**_4_) *G*(**r**_1_, **r**_2_, **r**_3_, **r**_4_)⟩ on a spatial grid with *N* point per dimension is being performed. This tensor contraction has *N*^12^ terms and a simple sequential evaluation will require *N*^12^ terms. For the stratified stochastic enumeration approach, we will first define a composite index *K* such that *K* = 1, ..., *N*^12^. The composite index, *K*, uniquely maps each ordered set of indices (*i*_1_, *i*_2_, *i*_3_, ..., *i*_12_) to an integer in 1, ..., *N*^12^.
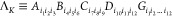
7Using the composite index *K*, we can define the summation as displayed in the following equation.
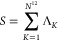
8Next, we divide the entire range of *K* into *N* nonoverlapping segments *N*_seg_ = *N*. The number of terms in each segment is *N*_*T*_ = *N*^11^. The summation over *K* can be written in terms of the segmented summation.
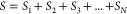
9
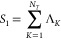
10
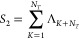
11
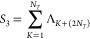
12
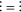
13
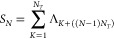
14The partial averages are defined as follows.
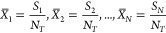
15The total sum can be written as displayed in the equation below.
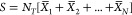
16In SSE, the sequential segment average, *X̅*, is approximated using the stochastic average,
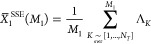
17
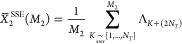
18
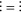
19
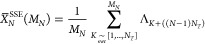
20where the subscript in  denotes “sampling-without-replacement”. For any segment “*p*”, the SSE average approaches the sequential average as *M*_*p*_ → *N*_*T*_.
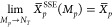
21The SSE estimate of the total summation is presented in the equation below.
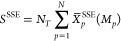
22The allocation of the sampling points for each segment is proportional to the variance in the SSE segment average *X̅*_*p*_^SSE^.
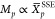
23
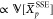
24The SSE approach is based on stratified sampling, which has been used extensively for reducing sampling error in Monte Carlo calculations^56−58^ and a brief description stratified sampling is presented in the Supporting Information (SI). The SSE method is not restricted to square tensors and can be applied to rectangular tensors as well. We recommend a row-major composite indexing scheme. For an index vector (*i*_1_, *i*_2_, *i*_3_, ..., *i*_*D*_), where *D* is the dimension of the tensor and each index (*i*_*d*_, *d* = 1, *D*) is in the range (*i*_*d*_ = 1, ..., *N*_*d*_), the row-major composite index *K* can be calculated using the following expression.
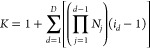
25

### 2.2 Second-Order Dyson Equation

In this work we are interested in calculating the IPs of chemical systems. The component of the 1-particle Green’s function, **G**(ω), that contains information about the IPs can be expressed in the Lehman representation as follows.^59^
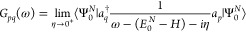
26where, *G*_*pq*_ is the matrix element of the matrix representation of the operator in canonical Hartree–Fock (HF) orbital basis {χ_*p*_},
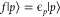
27{ *a*_*q*_^†^, *a*_*p*_,} are creation and annihilation operators defined with respect to the HF orbitals, {*E*_0_^*N*^, Ψ_0_^*N*^} are the exact ground state energies and wave function, and *H* is the electronic Hamiltonian. By inserting a complete set of projectors, 1 = ∑_*m*_ |Ψ_*m*_^*N*–1^⟩ ⟨Ψ_*m*_^*N*–1^|, we obtain the following equation.
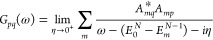
28As shown in eq 28, the Green’s function’s poles correspond to the vertical IPs of a many-electron system.
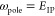
29The quantity |*A*_*mp*_|^2^ is the residue of the pole and is known as the pole strength.
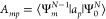
30The limit η → 0^+^ in eq 28 is traditionally associated with this expression because of its use in performing the Fourier transform from the time-domain to frequency-domain and will be suppressed in the rest of the derivation. Analogous to the many-body Green’s function, the uncorrelated HF Green’s function **G**^0^ is given by the following expression.^59^
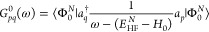
31which immediately simplifies to following diagonal representation.^59^
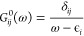
32where *i*, *j* = 1, *N*_occ_ are indices for occupied orbitals and ϵ_*i*_ is the orbital energy. The above equation recovers the Koopmans’ approximation to the IPs, which is defined as *E*_IP_^Koopmans^ = −ϵ_*i*_.

In the frequency representation, the relationship between the correlated 1-particle Green’s function, *G*, and the uncorrelated Green’s function *G*_0_ is given by the well-known Dyson equation.^60^
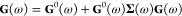
33Here, Σ is the self-energy operator. The relationship between the correlated and uncorrelated Green’s function can be derived using various techniques including time-dependent perturbation theory, time-independent perturbation theory, coupled-cluster theory, the configuration interaction method, and electron-propagator methods.^26,34,59,61,62^ The above operator equation can also be presented in various representations such as plane-waves, real-space grids, and canonical HF orbitals.^42,63,64^ In this work, we use canonical HF orbitals to represent the Dyson equation.^60^ To facilitate the calculation of the poles, it is useful to express eq 33 in terms of inverse operators by multiplying *G*^–1^(ω) from left and *G*_0_^–1^(ω) from the right.
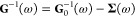
34Once **G**(ω) is determined for an appropriate set of values of ω, the poles can be observed by constructing a plot of **G**(ω) versus ω. Approximating the total self-energy operator by diagonal representation,
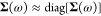
35allows for analytical inversion of the Dyson equation into the following simplified expression,^60^
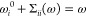
36where ω_*i*_^0^ is the orbital energy of occupied orbital (*i*) and ω = −*E*_IP_. We have used the second-order approximation to the self-energy operators, which in the canonical MO basis is defined as,^60^
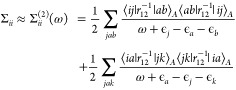
37where *i*, *j*, and *k* indicate occupied spin orbitals and *a* and *b* indicate virtual spin orbitals.

Using the restricted Hartree–Fock (RHF) formulation, the correction to the orbital energies using the second-order self-energy expression can be written as,
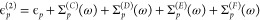
38where the RHF expressions for the self-energy terms are defined as,
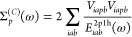
39
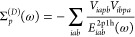
40
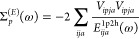
41
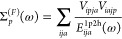
42Here, we have used the following compact notation for the energy denominators,
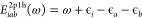
43
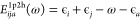
44and the *r*_12_^–1^ matrix elements are defined using the chemist’s notation for the indices.
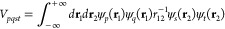
45Next, we will develop the SSE-MO approach for evaluating the self-energy operator.

### 2.3 Stratified Stochastic Enumeration of Self-Energy

We begin by defining a set  of ordered integers (*i*, *a*, *b*),
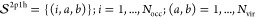
46which contains all the possible combinations of indices that occur in 2p1h self-energy expression. We will use the composite index *K* = (*i*, *a*, *b*) to enumerate this ordered set of integers. The size of set  is given as,
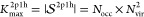
47Using this notation, we can define a general form of the 2p1h self-energy term as follows.
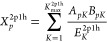
48where,
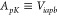
49
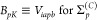
50
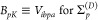
51In eq 48 the summation is performed sequentially for all terms. In the stochastic enumeration (SE) approach the sequential sum is replaced by a stochastic summation. We define a new operator Σ̃ which is defined as follows,
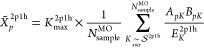
52and where *K* is sampled from the set . This sampling is performed without replacement and the notation  is used to emphasize this procedure (sample-without-replacement). The *N*_sample_^MO^ is sample size and bounded from above by *K*_max_^2p1h^. In the limit when the sample size approaches *K*_max_^2p1h^ the following limiting condition is satisfied.
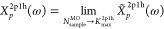
53Simple stochastic enumeration will involve performing the sampling over multiple runs and averaging the final results.
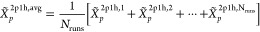
54The variance is defined as follows.
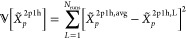
55We expect the variance to disappear when *N*_sample_^MO^ approaches *K*_max_^2p1h^,
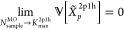
56To reduce the variance of the overall calculations, we introduce stratification in the sampling procedure. This is achieved in two steps. First, the set  is decomposed into a union of nonintersecting subsets,
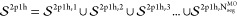
57
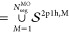
58where the subsets are nonoverlapping.
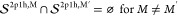
59The number of elements in subset M is denoted as *K*_max_^2p1h,M^.
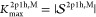
60In the second step, *X̃*^2*p*1*h*^ is calculated using summation over all the subsets.
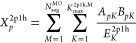
61The stratified stochastic enumeration of the MO indices (SSE-MO), which uses stochastic enumeration for segment sampling, is described by the following equation.
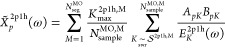
62To write the expressions in compact notation, we introduce the following for stochastic summation.
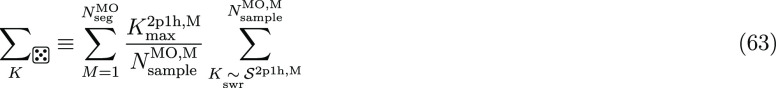
63Using this notation, we can write the following expression.
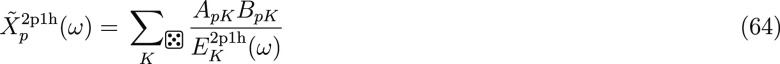
64A similar treatment is performed for the 1p2h terms. The combined result for the total self-energy operator is given as,
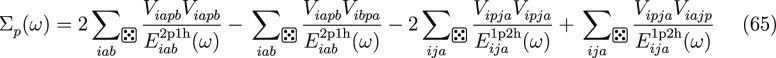
65

### 2.4 Calculation of Optimal Sampling Points for MO-Space Stratified Sampling

To calculate optimal sampling points, we define the segment average as,
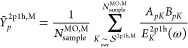
66which allows us to write the following expression.
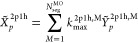
67It is important to note that *Ỹ*_*p*_^2p1h,M^ is a stochastic variable for which the average value, *Ỹ*_*p*_^2p1h,M,avg^, can be obtained by sampling over multiple runs.
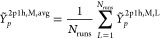
68The variance is defined as,
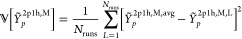
69The variance of *Ỹ* goes to zero as *N*_sample_^MO,M^ approaches *K*_max_^2p1h,M^,
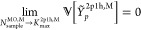
70To distribute the sampling points optimally, we define the following weight factor,
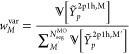
71In addition to that we also define another weight factor that depends on the magnitude of the terms,
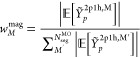
72The sampling is performed in batches, and the variance is updated after completion of a batch. The number of sampling points for each segment has the general form of,
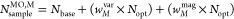
73where all segments get *N*_base_ sample points per batch irrespective of the segment average and variance. *N*_opt_ indicates the number of additional sample points that are distributed in a manner that is proportional to the normalized weights, which depend on the segment average and variance.

### 2.5 Low-Discrepancy Sampling without Replacement Using Quasi-Monte Carlo Method

It is important to note that obtaining  is a correlated sampling process. It is intrinsically non-Markovian and depends on the entire history of the string of previously generated indices. One way to achieve this in discrete integer space is by performing self-avoiding random walks. However, sampling in the self-avoiding random walker is local in nature, and therefore is not ideal for variance reduction in each segment. Here we use quasi-Monte Carlo sampling and a low-discrepancy integer sequence to perform sampling within each segment.^56−58^ The linear congruent generator for low-discrepancy quasi-random numbers is modified for generation of integer sequences.^56^ The sampling index for a segment *M* is defined as *K*^(*M*)^ and can have values in the range [1, ..., *K*_max_^(*M*)^]. The exact value of *K*_max_^(*M*)^ for each segment is known at the start of the calculation and is a consequence of the stratification procedure described in subsection 2.3. Associated with each segment are two integer random numbers which we define as *q*^(*M*)^ and *r*^(*M*)^. The variable *q*^(*M*)^ impacts the discrepancy of the points and is an integer random number chosen randomly from the interval *q*^(*M*)^ ∼ [10, 50]. Using *q*^(*M*)^, we define the following sequence from which *r*^(*M*)^ is selected randomly.
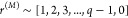
74Using *q*^(*M*)^ and *r*^(*M*)^, the low-discrepancy sequence is defined as,
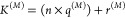
75
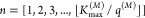
76where  is the floor of the ratio.

### 2.6 Control Variate for Monte Carlo Evaluation of Two-Electron Integrals

In this section, we extend the stochastic procedure developed in the previous section for numerical evaluation of the two-electron integrals. Monte Carlo evaluation of the two-electron integrals is not a requirement for implementing the SSE-MO method, and the SSE-MO procedure described in section 2.3 can be used whenever the two-electron repulsion integrals, *V*_*pqst*_, are available in MO representation. However, for large systems, it is computationally advantageous to avoid the AO-to-MO two-electron transformation and instead, to numerically integrate directly in the MO representation using the Monte Carlo scheme.

Associated with each MO pair function, ψ_*p*_(**r**)ψ_*q*_(**r**), we define a control-variate function, ψ_*pq*_^*cv*^(**r**). The control variate function must satisfy two important features. First, ψ_*pq*_^*cv*^(**r**) must be nonfactorizable as a product of functions that depend only *p* and *q* indices.
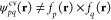
77Second, the two-electron integrals, [ψ_*pq*_^cv^(**r**_1_)|*r*_12_^–1^|ψ_*st*_^cv^(**r**_2_)], must be known analytically. Adding and subtracting the control variate function, we express the MO product function as follows.
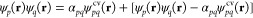
78
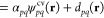
79Here, α_*pq*_ is the control variate and *d*_*pq*_(**r**) is the difference function. Using the above expression, the two-electron integral can be expressed as,
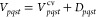
80where,
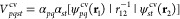
81and,
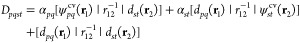
82The control variate, α_*pq*_, is defined as the quantity that minimizes the following weighted-variance function.
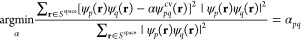
83Here, *S*^space^ is a set of sampling points in 3D space from which **r** is drawn at random. The control-variate functions, ψ_*pq*_^cv^, are represented by Gaussian functions. For *p* = *q*, a single Gaussian function is used and for *p* ≠ *q* a linear combination of two Gaussian functions is used.
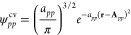
84
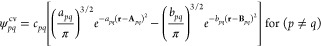
85This form of the control variate function guarantees the orthonormality conditions for the MOs.
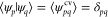
86The widths and the centers of the Gaussian functions are determined using a moment-matching condition. The weighted moments for any pair of molecular orbitals are calculated as,
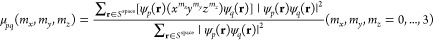
87and the moments for the control-variate functions are obtained analytically.
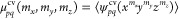
88The coefficients for the control-variate function are obtained by performing a steepest-descent search on the following loss-function.
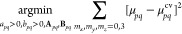
89The equations eq 83 and eq 89 completely define the control-variate function and are used to calculate the *V*_*pqst*_^cv^ term.

### 2.7 Monte Carlo Evaluation of Two-Electron Integrals Using Real-Space Stratified Sampling

Since *V*_*pqst*_^cv^ is analytical, only the *D*_*pqst*_ terms are calculated numerically using the stratified Monte Carlo procedure. We use a combination of ratio estimator, control variate, and stratified sampling techniques to efficiently and accurately evaluate the MO integrals. For calculating *D*_*pqst*_, we define the following two-electron kernel function,
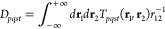
90where,
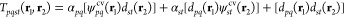
91Associated with each *D*_*pqst*_ integral, we define a control variate *f*_*pqst*_^cv^ as,
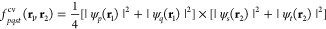
92where the integral of the control variate is 1 for all values of *p*, *q*, *s*, *t*.
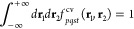
93The calculation of *D*_*pqst*_ requires evaluation of the six-dimensional integral over all space. Traditionally, Monte Carlo integration is performed over an *N*-dimensional unit cube by transforming the integral range from [−∞, + ∞] to [0, 1]. One approach to achieve this is by using the following transformation,
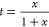
94
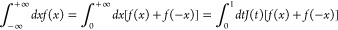
95However, this procedure introduces singularity in the form of the Jacobian *J*(*t*) in the integration kernel. In this work, we use a finite-grid approximation to evaluate the integral over a finite volume,
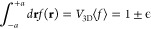
96where the limits selected are large enough so that ϵ ≤ 10^–5^ for all MOs. Using *f*_*pqst*_^cv^, we define the following ratio estimator for Monte Carlo evaluation of the *D*_*pqst*_ integral.
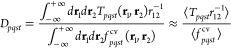
97The averages ⟨*T*_*pqst*_*r*_12_^–1^⟩ and ⟨*f*_*pqst*_^cv^⟩ are defined as follows.
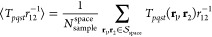
98
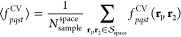
99We introduce stratification in the sampling of points in real-space by dividing the entire space into a set of *N*_seg_^space^ nonoverlapping regions with identical volumes.
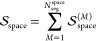
100
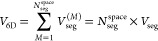
101The stratified sampling estimate of the averages is then defined as,
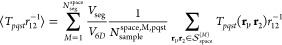
102*N*_sample_^space,M,pqst^ is the number of sampling points associated with the spatial segment, *M* for indices *p*, *q*, *s*, *t*. Similar to the stratification strategy in eq 73, the sampling points for each segment are proportional to the variance of the integral kernel in that segment.
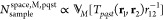
103
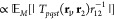
104We use common-random-number (CRN) sampling for sampling within a segment. The CRN method has been used extensively for reducing variance^56−58^ and a brief summary of the method is presented in ref (65). In the evaluation of the integral, this means that at any point in time, if a random number η_1_ is used in the evaluation of *T*_*pqst*_, then that same random number is used for evaluation of *f*_*pqst*_^cv^ in the same segment. To emphasize this usage, we use superscript “CRN” (common random number) in the following expression.
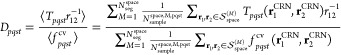
105

### 2.8 Iterative Solution of the Dyson Equation

Combining the results from section 2.3 and section 2.7, we can express the full self-energy operator as the sum of two terms,
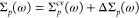
106The first term Σ_*p*_^cv^(ω) depends only on control-variate functions and is evaluated analytically.
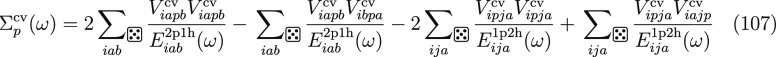
107The second term contains the different terms and is defined as follows.
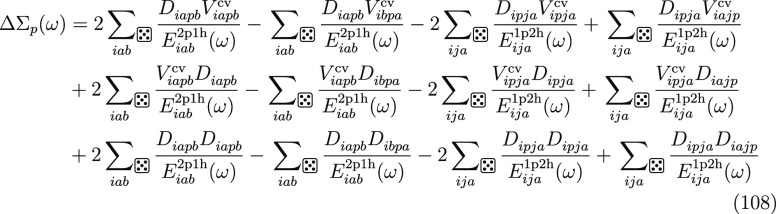
108

The single-shot determination of the self-energy operator is performed by evaluating the self-energy at the HOMO energy.
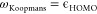
109
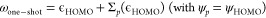
110The full iterative solution of the Dyson equation is obtained by evaluating Σ_*p*_(ω) for a range of ω and then finding the point where,
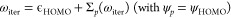
111The SSE-MO method allows for a third approximation for ω. We can solve the Dyson equation iteratively using Σ_*p*_^cv^(ω)
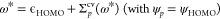
112and then include the correction from Δ*Σ*_*p*_.
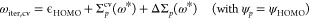
113

### 2.9 Calculation of Derivatives

The first derivative of the self-energy, with respect to ω, is useful for locating the poles of **G**(ω), and is defined as follows.
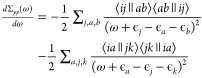
114The higher-order derivatives of the self-energy operator can then be obtained from the higher-order powers of the denominator.
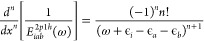
115
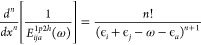
116In the SSE-MO method, the derivative of Σ_*p*_ is obtained by replacing the energy denominator with the higher powers of the denominator,
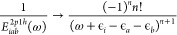
117
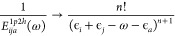
118The contributions from Σ_*p*_^cv^ are obtained analytically, and the contributions from the Δ*Σ*_*p*_ are obtained from the stochastic enumeration procedure described above. Because the derivative does not impact *r*_12_^–1^, which is present in expression for the self-energy, the calculation of the derivatives can be performed concurrently while the calculation of Δ*Σ*_*p*_ is being performed. This approach was used for the construction of the spectral function and is presented in section 3.2.

### 2.10 Computational Details

The SSE-MO method was applied to investigate the ionzation potentials of PbS and CdS QDs. In addition, benchmark calculations for Ne, H_2_O, and CH_4_ were also performed. The single-particle states and energies were obtained from HF calculations using the 6-31G* basis for Ne, H_2_O, and CH_4_ and the LANL-2DZ ECP basis for the quantum dots. These HF calculations were performed using the TERACHEM electronic structure package. The SSE-MO calculations were performed by dividing the MO-index space into *N*_occ_ number of segments. The 6D Cartesian space was divided into 100 nonoverlapping regions. A total of *N*_sample_ ∼ 10^9^ sampling points were used for calculating the self-energy at each value of ω and the sampling points were distributed using the stratification strategy described earlier. We used the relative standard deviation, σ_rel_, also known as coefficient of variance for defining the convergence criteria for the calculated IPs (eq 119).
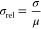
119In this work, we enforced σ_rel_ < 10^–2^ to be the criteria for convergence for each segment.

## 3 Results

### 3.1 10-Electron System

For benchmarking and testing, the SSE-MO method was used to calculate the IPs of Ne, H_2_O, and CH_4_. The results for these chemical systems are presented in Table 1. The IPs were calculated using both single-shot and iterative solution of the Dyson equation and the results between the two approaches were found to be very similar to a maximum difference of 0.17 eV. In all cases, the SSE-MO results were found to be in good agreement with the previously reported results.

**Table 1:** Ionization Potentials (eV) of Ten Electron Systems: Comparison with Benchmark Literature Values

system	Koopmans’	single-shot solution	iterative solution	lit. value^66,67^	IP-EOM-CCSD(T)^68^
CH_4_	14.86	13.94 ± 0.03	13.95 ± 0.03	13.91	12.76
Ne	22.59	21.47 ± 0.06	21.38 ± 0.07	21.13	20.98
H_2_O	13.56	10.44 ± 0.13	10.61 ± 0.08	10.74	11.37

### 3.2 Ionization Potential of PbS and CdS Quantum Dots

**Figure 2:**
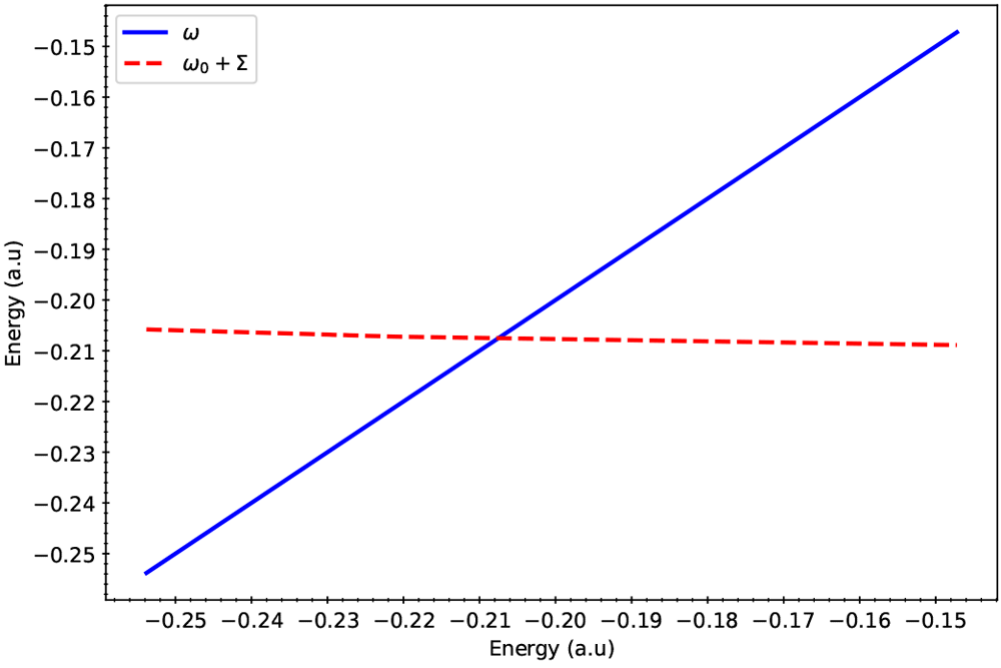
ω (ordinate) versus ω (abscissa) displayed as the curve labeled ω. The curve labeled ω_0_+Σ displays the relationship between the HOMO energy + the self-energy (ordinate) and ω. The value of ω at which these two curves intersect is equivalent to the value of ω for which the diagonal approximation to the Dyson equation converges.

**Figure 3:**
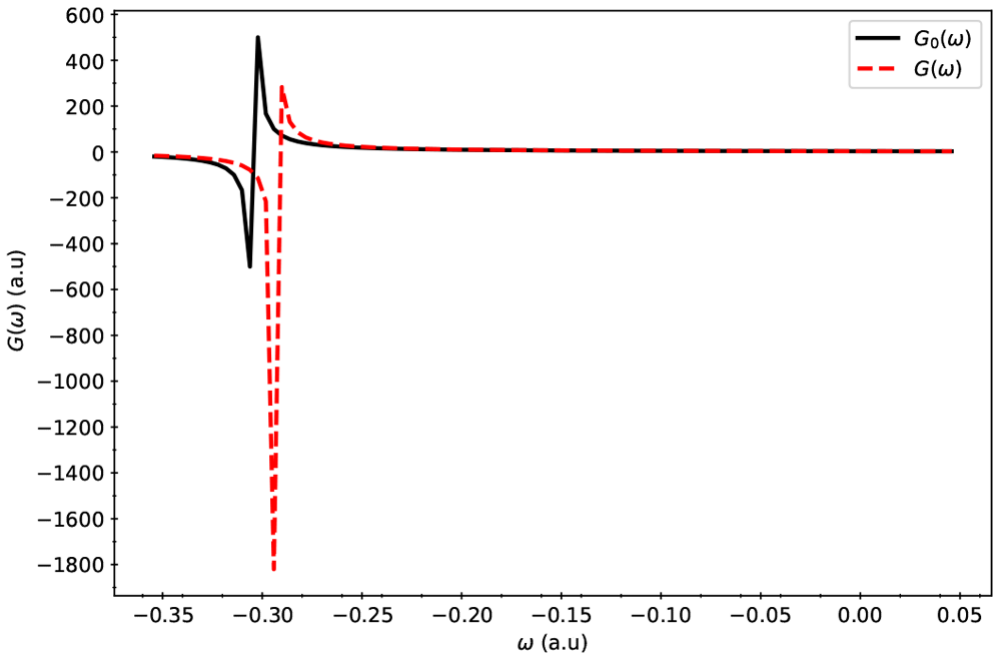
Poles of *G*_0_(ω) and *G*(ω) for the Pb_4_S_4_ system. *G*(ω) (ordinate) versus ω (abscissa) is labeled as *G*(ω) in the legend. The curve labeled *G*_0_(ω) displays the relationship between *G*_0_(ω) (ordinate) and ω (abscissa).

**Figure 4:**
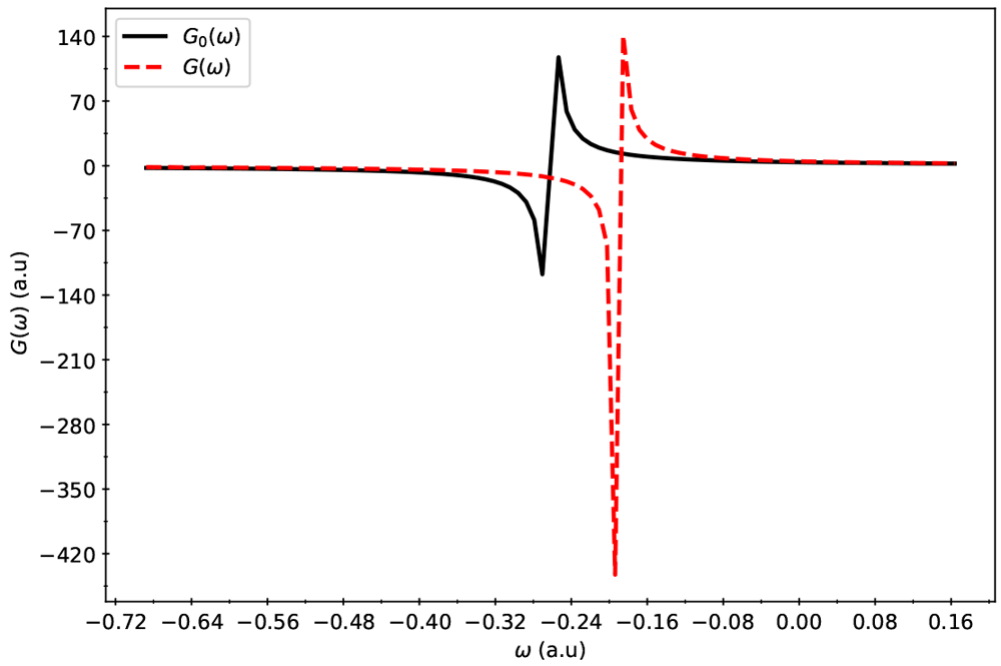
Poles of *G*_0_(ω) and *G*(ω) for the Pb_44_S_44_ system. *G*(ω) (ordinate) versus ω (abscissa) is labeled as *G*(ω) in the legend. The curve labeled *G*_0_(ω) displays the relationship between *G*_0_(ω) (ordinate) and ω (abscissa).

**Figure 5:**
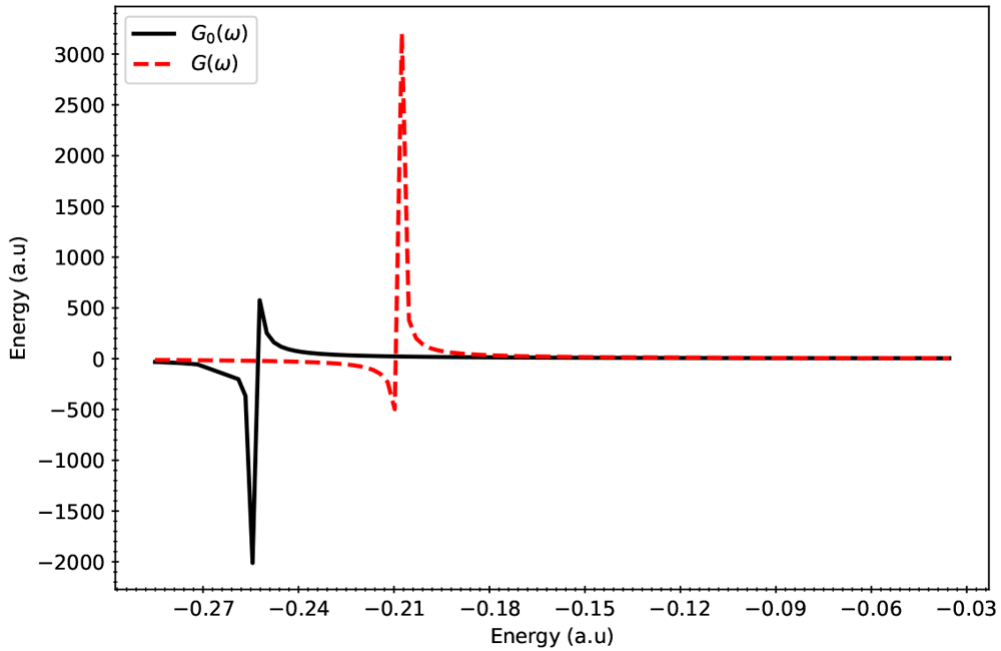
Poles of *G*_0_(ω) and *G*(ω) for the Pb_140_S_140_ system. *G*(ω) (ordinate) versus ω (abscissa) is labeled as *G*(ω) in the legend. The curve labeled *G*_0_(ω) displays the relationship between *G*_0_(ω) (ordinate) and ω (abscissa).

The SSE-MO method was applied to Pb_4_S_4_, Pb_44_S_44_, Pb_140_S_140_, Cd_6_S_6_, Cd_24_S_24_, and Cd_45_S_45_ and the ionization potentials from the single-shot and iterative solution of the Dyson equation are presented in Table 2 and Table 3, respectively. We note that the calculated IPs are vertical ionization potentials and do not include contributions from the quantum mechanical treatment of nuclear degrees of freedom. Figure 2 illustrates the graphical verification of the self-consistency of the iterative procedure for Pb_140_S_140_. We observe that the curve for Σ(ω) + ω_0_ versus ω intersects with the curve for ω versus ω at that the value of ω for which the diagonal approximation to the Dyson equation converges. The frequency dependence of the 1-particle Green’s function was evaluated near the poles and is presented in Figures 3, 4, and 5.

**Table 2:** Self-Energy and Ionization Potentials (eV) of PbS and CdS Quantum Dots from Single-Shot Solution

system	Koopmans’	self-energy from single-shot solution	IP from single-shot solution
Pb_4_S_4_	8.28	0.65	7.63 ± 0.05
Pb_44_S_44_	7.13	0.22	6.91 ± 0.04
Pb_140_S_140_	6.91	0.09	6.82 ± 0.05
Cd_6_S_6_	5.25	0.42	4.837 ± 0.04
Cd_24_S_24_	6.25	0.09	6.16 ± ϵ < 0.01
Cd_45_S_45_	6.09	0.23	5.86 ± 0.02

**Table 3:** Self-Energy and Ionization Potentials (eV) of PbS and CdS Quantum Dots from Iterative Solution

system	Koopmans’	self-energy from iterative solution	IP from iterative solution
Pb_4_S_4_	8.28	0.61	7.66 ± 0.01
Pb_44_S_44_	7.13	0.17	6.96 ± 0.01
Pb_140_S_140_	6.91	0.28	6.71 ± 0.08
Cd_6_S_6_	5.25	0.41	4.84 ± 0.01
Cd_24_S_24_	6.25	0.08	6.16 ± ϵ < 0.01
Cd_45_S_45_	6.09	0.22	5.87 ± 0.01

When compared to *G*_0_(ω), the poles of *G*(ω) were found to have higher values of ω indicating that for these systems, inclusion of electron correlation effects resulted in a lower IP than Koopmans’ IP values. Comparison between *G* and *G*_0_ shows that inclusion of electron correlation in IPs becomes more important for larger dots. Comparison between the single-shot versus iterative solution of Dyson equation also exhibits similar trends, where the need for iterative solutions become more important for larger dots.

### 3.3 Single-Pole Approximation to the Spectral Function

**Figure 6:**
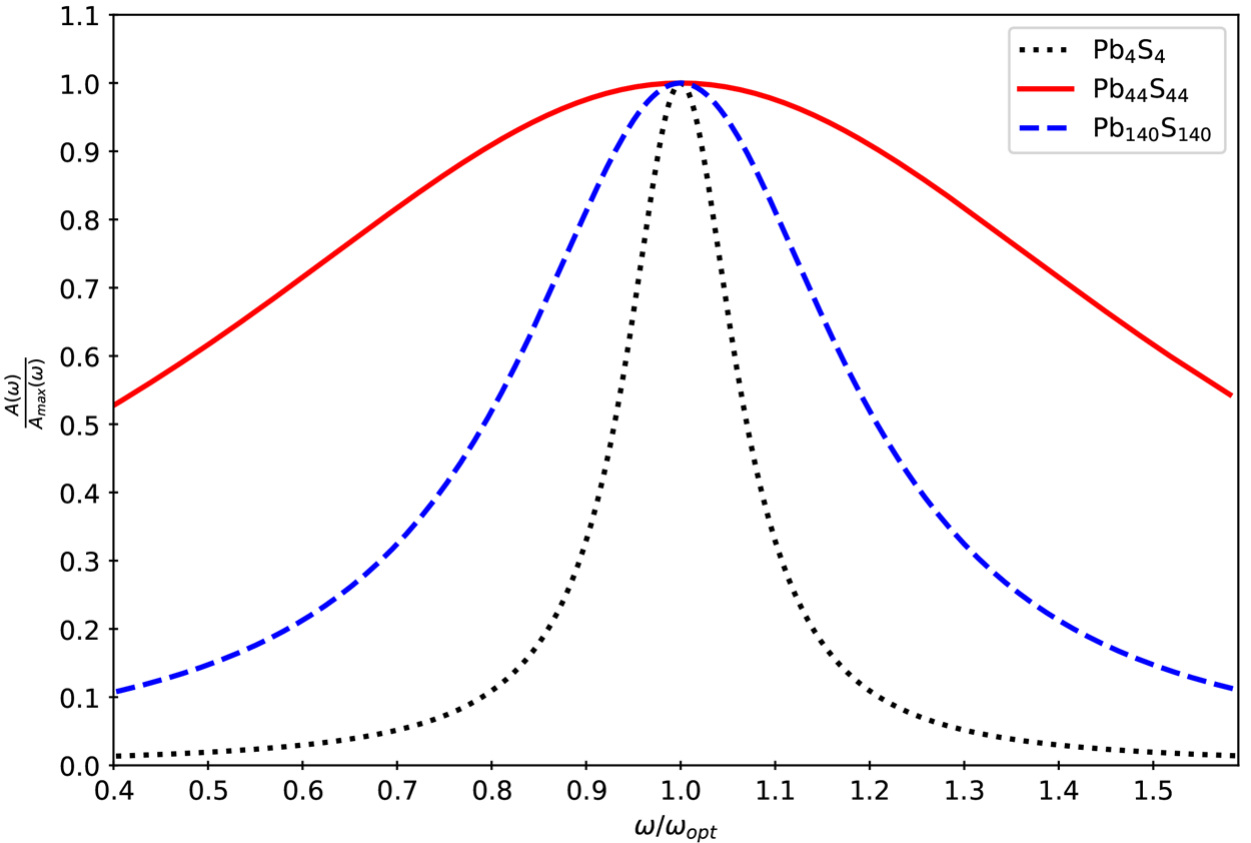
Ratio *A*_wp_/*A*_wp_^max^ (ordinate) plotted as a function of ω/ω_opt_ (abscissa) for a series of PbS quantum dots. *A*_wp_/*A*_wp_^max^ is the ratio of *A*_sp_(ω) and the maximum value of *A*_sp_(ω). ω_opt_ is the value of ω for which convergence of the diagonal approximation to the Dyson equation is achieved.

We define the single-pole approximation to the spectral function as,
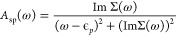
120where the subscript “sp” in *A*_sp_ denotes that we are looking at the form of the spectral function near the pole (ω= ϵ_*p*_). The imaginary part of the self-energy operator can be approximated from the first derivative of the self-energy, with respect to ω, as described in section 2.9. For example, the imaginary part of the following quantity *I*(ω),
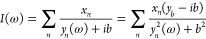
121is given by,
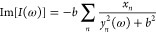
122In Figure 6, the ratio *A*_wp_/*A*_wp_^max^ is plotted as a function of ω/ω_opt_ for the three PbS QDs.

**Figure 7:**
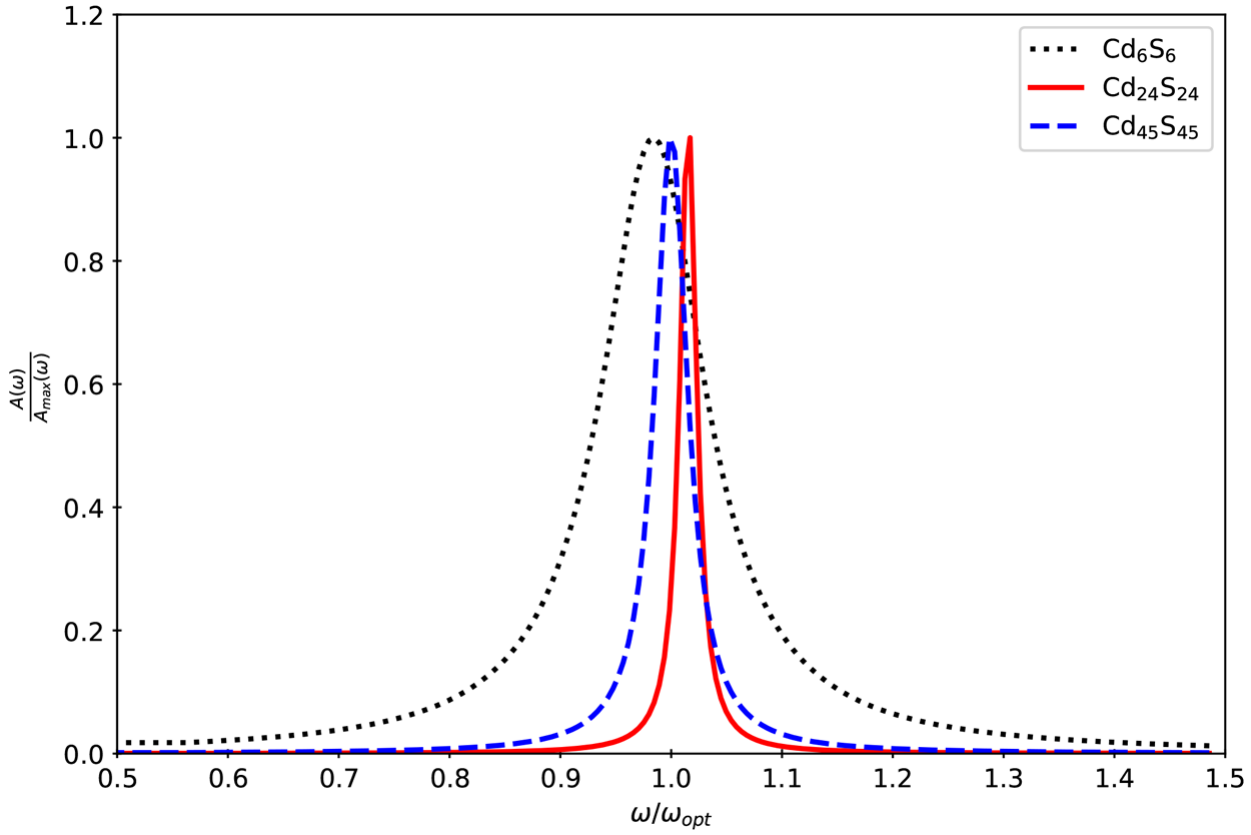
Ratio *A*_wp_/*A*_wp_^max^ (ordinate) plotted as a function of ω/ω_opt_ (abscissa) for a series of CdS quantum dots. *A*_wp_/*A*_wp_^max^ is the ratio of *A*_sp_(ω) and the maximum value of *A*_sp_(ω). ω_opt_ is the value of ω for which convergence of the diagonal approximation to the Dyson equation is achieved.

The line width of the plot was found to be narrowest for the Pb_4_S_4_ and broadest for Pb_44_S_44_. This feature indicates that the relative lifetime of the quasi-hole in the intermediate dot size (Pb_44_S_44_) is longer than the other dots in the series. Similar analysis for the CdS QDs in Figure 7 revealed that the line width decreases with increasing dot size. The results from the spectral analysis highlight the importance of including frequency dependency in the self-energy operator. The plots also demonstrate the impact of many-body correlations in the these systems. Specifically, in the absence of electron–electron correlation, the limit σ → 0 will reduce the plots to a Dirac delta function.

## 4 Discussion

### 4.1 Correlated Sampling in the Combined Cartesian and Molecular Orbital Index Space

The main philosophy of the SSE-MO method is to perform correlated sampling in a joint real-space and occupation-number space (Table 4). Assuming a discretization of 100 points per Cartesian coordinate, the total number of points needed for exhaustive sampling is in the range of 10^16^ to 10^20^ as shown in Table 4. However, not all spatial components of all the molecular orbitals contribute equally and uniformly to the calculation of the self-energy. There are certain combinations of MOs whose form in specific regions of the Cartesian space correlate strongly with the error in the self-energy calculations. Through the use of a two-step stratified sampling scheme in both Cartesian and MO space, the SSE-MO method provides a systematic and adaptive procedure to identify the important contributors. We have used a combination of ratio estimator, control-variate, and stratified sampling techniques for the efficient and accurate evaluation of the MO integrals. The key quantity that implements and controls this concept is the *N*_sample_^space,M,pqst^ term. This term represents the number of spatial sampling points for the Mth spatial segment for the correction term *D*_*pqrs*_ associated with indices *p*, *q*, *r*, *s* and depends on both the spatial and MO indices. The total number of sampling points is given by the following expression.
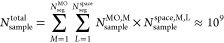
123As shown in eq 103, this number was directly obtained from the variance of the integral kernel, which also includes the contribution from the *r*_12_^–1^ operator. Note that these sampling points were not used to evaluate the full r_12_-integral kernel, but instead were used to evaluate only the component of the full r_12_-integral kernel not included in the control-variate expression. The Cartesian space sampling for each spatial segment was performed using simple Monte Carlo sampling. This process can be enhanced by using low-discrepancy random numbers, which is a quasi-Monte Carlo approach. We expect that using the quasi-Monte Carlo approach will accelerate the overall calculation process.

**Table 4:** Total Number of Sampling Points in the Combined MO-Cartesian Space Assuming 100 Points Per Cartesian Coordinate

system	*N*_2p1h_	*N*_1p2h_	*N*_space_^MO^	*N*_space_^MO^ × *N*_space_^6D^
Pb_4_S_4_	3.87 × 10^4^	1.76 × 10^4^	5.63 × 10^4^	5.63 × 10^16^
Pb_44_S_44_	5.15 × 10^7^	2.34 × 10^7^	7.50 × 10^7^	7.50 × 10^19^
Pb_140_S_140_	1.18 × 10^9^	6.37 × 10^8^	1.82 × 10^9^	1.82 × 10^21^
Cd_6_S_6_	7.02 × 10^5^	3.32 × 10^5^	1.03 × 10^6^	1.03 × 10^18^
Cd_24_S_24_	4.50 × 10^7^	2.13 × 10^7^	6.62 × 10^7^	6.62 × 10^19^
Cd_45_S_45_	2.96 × 10^8^	1.40 × 10^8^	4.36 × 10^8^	4.36 × 10^20^

### 4.2 Segment-Based Analysis of Sampling Error

**Figure 8:**
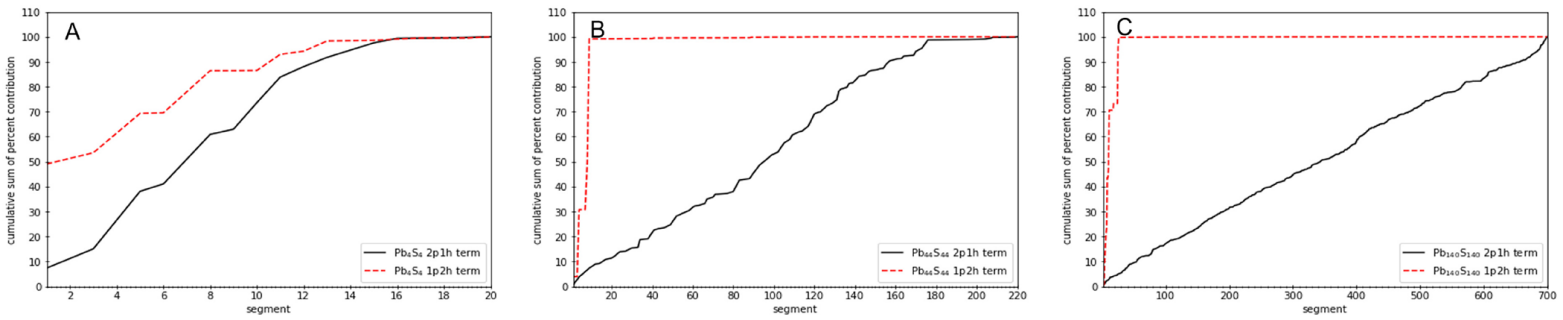
Cumulative sum of the percent contributions of the segments composing the sample space for the 2p1h and 1p2h terms of the self-energy (ordinate) versus the segment index (abscissa) is displayed. Parts A, B, and C, are for QDs Pb_4_S_4_, Pb_44_S_44_, and Pb_140_S_140_, respectively.

**Figure 9:**
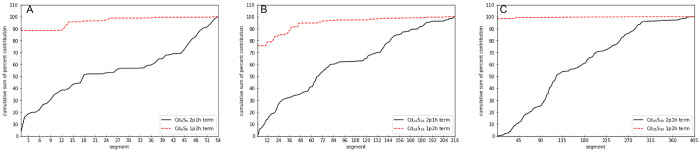
Cumulative sum of the percent contributions of the segments composing the sample space for the 2p1h and 1p2h terms of the self-energy (ordinate) versus the segment index (abscissa) is displayed. Parts A, B, C are for QDs Cd_6_S_6_, Cd_24_S_24_, and Cd_45_S_45_, respectively.

The error in the calculated IP using the SSE-MO method originates from the sampling error associated with sampling the integral kernel in the combined Cartesian-MO space. However, not all segments contribute equally to the numerical error. The goal of SSE-MO is to distribute the computational effort in proportion to the contributions from each segment. One insight generated from the SSE-MO calculation is information about the contribution of each segment to the total self-energy operator. We define the cumulative percent contribution for the segments as,
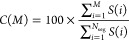
124where *S*(*i*) is the contribution to the self-energy for each segment. The cumulative percent contribution of the segments to the total self-energy operator is denoted as *C*(*M*) and is presented for the PbS and CdS quantum dots in Figures 8 and 9, respectively.

**Figure 10:**
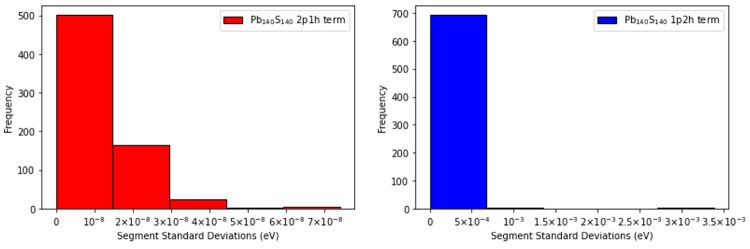
Frequency distributions of the standard deviations (in eV) for the segments that compose the sample space of the 2p1h and 1p2h terms of the self-energy computed for Pb_140_S_140_, are displayed.

**Figure 11:**
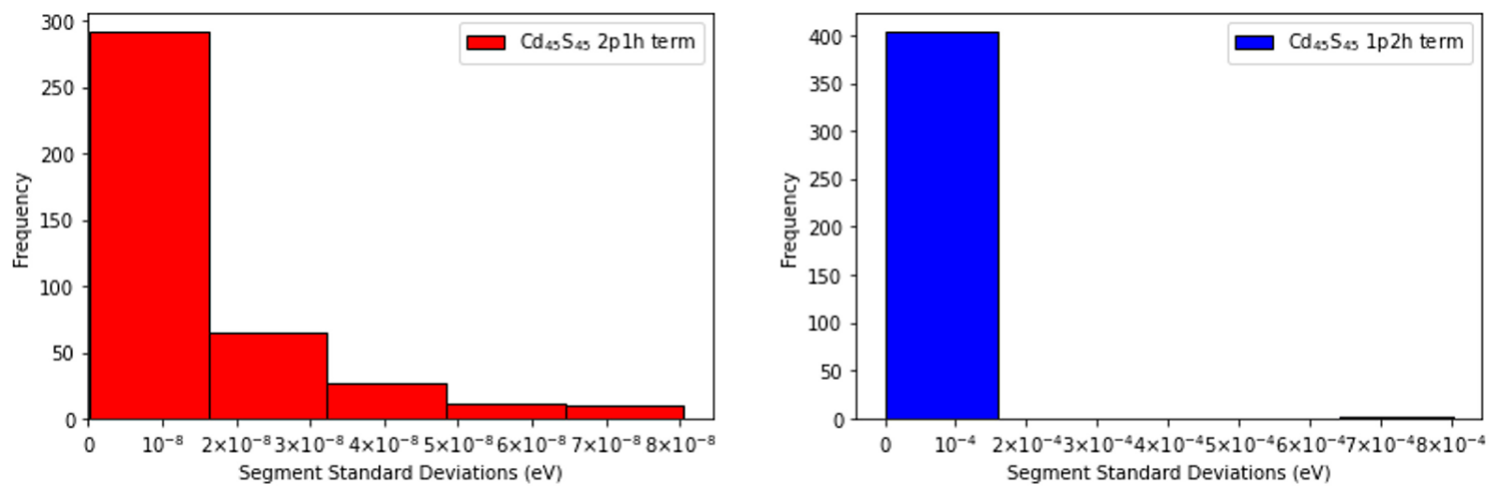
Frequency distributions of the standard deviations (in eV) for the segments that compose the sample space of the 2p1h and 1p2h terms of the self-energy computed for Cd_45_S_45_, are displayed.

Analysis of the results revealed that the 2p1h and 1p2h terms show very different behavior. In all cases it was found that only few segments, typically ≤50, had significant contributions to the 1p2h component of the self-energy operator. In contrast, for the 2p1h component, the cumulative sum of the percent contribution increased in a much more gradual manner. The distributions of the standard deviations associated with the segments for the 2p1h and 1p2h terms of the self-energy for the two largest quantum dots, (Pb_140_S_140_ and Cd_45_S_45_), are presented in Figures 10 and 11, respectively.

**Figure 12:**
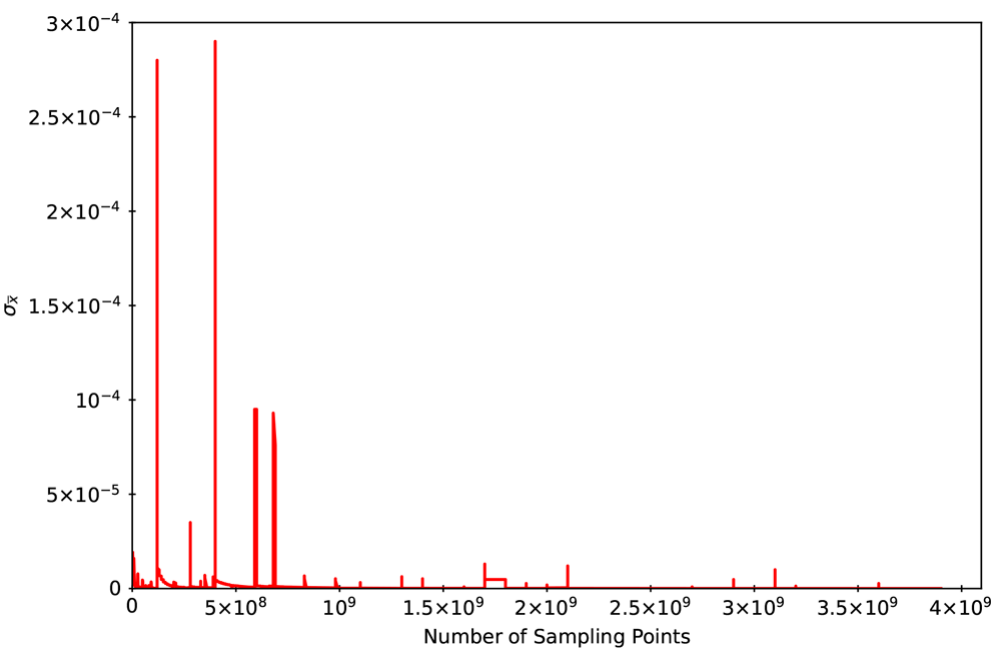
Sample standard deviation (σ _*x̅*_) in the ionization potential (eV) (ordinate) versus the number of sampling points (abscissa) used to construct the self-energy for the Pb_140_S_140_ dot is displayed.

Analysis of the distributions reveals that the sampling error in the 2p1h term is significantly smaller than the sampling error in the 1p2h term for the two largest quantum dots. These plots also show that the overall sampling error in the calculated IP is dominated by the sampling error in the 1p2h term. The advantage of the SSE-MO method is that, by construction, the SSE-MO scheme is able to extract this information dynamically during the course of the calculation and then allocate more sampling points to segments that have high sampling errors. Because SSE-MO is based on stratified sampling, the conventional stratified sampling error analysis^69^ is applicable for the sampling error in the IP calculations. In addition to this segment-based analysis, the overall sampling error in the calculated IPs as a function of the number of sampling points used to construct the self-energy for Pb_140_S_140_ quantum dot is presented in Figure 12.

### 4.3 Connection with Diagrammatic Monte Carlo

The SSE-MO method is conceptually similar to diagrammatic MC (diagMC), where terms are evaluated stochastically. However, there are key differences between the two methods. SSE-MO is not diagram-based and the relative importance of the terms are not evaluated using topological connectivity of the vertices. Also, the SSE-MO method uses stratified sampling as opposed to importance sampling, where emphasis is placed on reducing numerical error through variance minimization and numerical effort is predominantly spent on computing the correction term to the self-energy operator. As an intrinsically adaptive approach, the calculation puts more points where they are needed to achieve reduction of numerical error.

#### 4.3.1 Comparison with Laplace-Transformed Approach

The SSE-MO method does not perform Laplace-transformation, but instead relies on stochastic enumeration to reduce the computational cost. Consequently, only 3D and 6D spatial integrals are solved numerically. As a consequence, higher-order derivatives of the self-energy operator (*d*^*n*^Σ/*dω*^*n*^) can be obtained with relative ease and with very little additional computational cost during the self-energy calculation. This not only allows for calculation of the imaginary component of the self-energy operator, but also open doors for iterative solution of the Dyson equation by Taylor-series expansion of the self-energy operator.
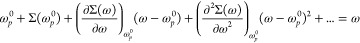
125Because we are not using a Laplace transformation, the SSE-MO method is well-suited to extending the self-energy calculation to Σ^(3)^ using the P3 correction developed by Ortiz and co-workers.^67^ For example, the Laplace transformation of the following term in the P3 expression
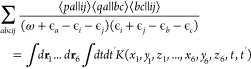
126will involve the Monte Carlo numerical integration of a 20-dimensional integral. In the SSE-MO implementation, the dimensionality of the spatial integral will still be six and the MO index will be sampled from the 3p-2h space.
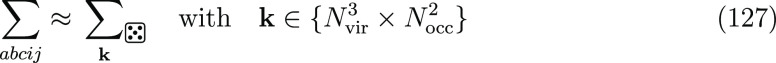
127Equation 127 can be viewed as the stochastic tensor contraction over the MO indices and can potentially be applied to other branches of quantum mechanics.

### 4.4 Selection of Control-Variate Functions

The use of moment-based fitting ensures that the integral of the *M*^th^-order multinomial comes out to be exact. For this work, a maximum of two Gaussian functions were used and was found to be adequate. For more challenging systems, the number of Gaussian functions can be systematically increased. In addition, metrics other than the moments can be used as criteria for the selection of the Gaussian functions. The choice of the control-variate functions is not restricted to Gaussian functions. For QDs, it is possible to take advantage of the approximate spherical symmetry of the system and construct the control-variate functions from the hydrogenic wave functions with effective hole and particle masses.
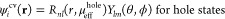
128
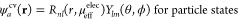
129

Although both density fitting^70^ and the control variate schemes use Gaussian functions, their purpose and implementation are very different. When using the control variate scheme, the goal is to reduce numerical error. When using density fitting, the goal is to approximate it. Specifically in the control variate, the integral of *f*_*pq*_ is expressed as
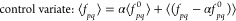
130
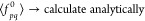
131
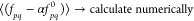
132
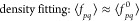
133

There are two main differences between control variate and density fitting:

1. When using the control variate scheme, the error in fitting the integral is always calculated. The error in the estimation of the integral comes from the numerical approximation to the analytical fitting error. If we were to replace the numerical integral, ⟨(*f*–*f*_0_)⟩, by an analytical integral, we would recover the exact integral. The origin of error in density fitting comes from the finite expansion of the auxiliary basis. While in the control variate the error is from the numerical approximation to the residue-error integral, ⟨(*f*–*f*_0_)⟩.
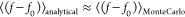
134
2. Unlike density-fitting’s attribute of fit-once-use-everywhere, the control variate approach is kernel dependent. This means that the integrals ⟨*f*_*pq*_*K*_*A*_⟩ and ⟨*f*_*pq*_*K*_*B*_⟩ will have different control variate parameters α_*A*_ and α_*B*_, respectively. These parameters are obtained by minimizing the variance as shown below:
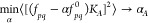
135
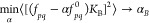
136One approach to do the above integrals efficiently is to first expand the square term and then perform the α-independent integrals separately as shown below.
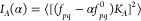
137
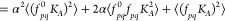
138

## 5 Conclusions

This work presents the development and implementation of the stratified stochastic enumeration of molecular orbitals (SSE-MO) method for construction of the self-energy operator. The central idea of this method is to express the self-energy operator in a composite space, which is generated by combining the 3D Cartesian space of molecular orbitals with the discrete integer space of the molecular orbital indices. In conjunction, a stratified sampling Monte Carlo scheme was also developed for the efficient evaluation of the complex self-energy operator and its frequency derivatives. The SSE-MO method was applied to a series of CdS and PbS QDs, and the IPs of these QDs were obtained from both single-shot and iterative solution of the second order diagonal approximation to the Dyson equation. The results from these calculations showed that the IPs decreased with increasing dot size. The imaginary component of the self-energy operator was used to construct the single-pole frequency-dependent spectral functions of the quantum dots. The quantum dots with the longest relative lifetimes of the quasi-hole state were identified. The strategy of stochastic enumeration used in the SSE-MO method can also be interpreted in the broader context of stochastic tensor contraction methods and can be applied to other areas of quantum mechanics, where the sequential enumeration of summations is computationally prohibitive.

## Supporting Information Available

Brief description of stratified sampling and common-random-number sampling (PDF)
