# Supplementary material for: Investigation of Ionization Potential in Quantum Dots Using the Stratified Stochastic Enumeration of Molecular Orbitals Method
Source: J Chem Theory Comput. 2022 Sep 22;18(10):5920–35. doi: 10.1021/acs.jctc.2c00329 (PMC9558315; doi:10.1021/acs.jctc.2c00329)
Supplement: Supplementary file 1 — ct2c00329_si_001.pdf [file ct2c00329_si_001.pdf]

Supporting Information for Investigation of Ionization Potential in Quantum Dots Using the Stratified Stochastic Enumeration of Molecular Orbitals method

Nicole Spanedda,[†] Peter F. McLaughlin,[†] Jessica J. Beyer,[‡] and Arindam Chakraborty[*,†]

† *Department of Chemistry, Syracuse University, Syracuse, New York 13244 USA*

‡ *Keck Science Department, Scripps College, Claremont, California, 91711, USA*

E-mail: archakra@syr.edu

A brief description of the stratified sampling and common random number sampling schemes is presented in this appendix. Both of these methods were used for variance reduction in the SSE-MO calculations performed.

Stratified Sampling

We start by considering a one-dimensional continuous function $f(x)$. The exact mean, μ_f, and the variance, σ_f^2, of this function is defined by the following limiting conditions.

$$\mu_f = \lim_{N\to\infty} \frac{1}{N} \sum_{i=1}^{N} f(x_i) \tag{1}$$

$$\sigma_f^2 = \lim_{N\to\infty} \frac{1}{N} \sum_{i=1}^{N} [f(x_i) - \mu_f]^2 \tag{2}$$

The quantity $\langle f \rangle$ is a finite sample estimator of μ_f,

$$\langle f \rangle = \frac{1}{N} \sum_{i=1}^{N} f(x_i) \tag{3}$$

$$\lim_{N \to \infty} \langle f \rangle = \mu_f \tag{4}$$

and the variance in the estimator of the mean, μ_f, is given as follows.

$$\mathbb{V}[\langle f \rangle] = \frac{\sigma_f^2}{N} \tag{5}$$

In stratified sampling, we divide the entire region into equal non-overlapping regions labeled as "A" and "B", respectively.

$$\langle f_A \rangle = \frac{1}{N_A} \sum_{i \in A} f(i) \tag{6}$$

$$\langle f_B \rangle = \frac{1}{N_B} \sum_{i \in B} f(i) \tag{7}$$

The sample average of the entire region can be obtained using the averages of the two regions, as displayed in the equation below.

$$\langle f \rangle = \frac{1}{2} \langle f_A \rangle + \frac{1}{2} \langle f_B \rangle \tag{8}$$

The variance is given by the following equation.

$$\mathbb{V}[\langle f \rangle] = \frac{1}{4} \mathbb{V}[\langle f_A \rangle] + \frac{1}{4} \mathbb{V}[\langle f_B \rangle] \tag{9}$$

The variance can be expressed in terms of σ_A^2 and σ_B^2.

$$\mathbb{V}[\langle f \rangle] = \frac{1}{4} \frac{\sigma_A^2}{N_A} + \frac{1}{4} \frac{\sigma_B^2}{N_B} \tag{10}$$

Equation 10 shows that the variance of the sample mean can be minimized by using differing numbers of sampling points for the regions, A and B. The extension of this approach for more than two segments is described in books on Monte Carlo sampling.[1,2] It can be shown that the variance obtained using stratified sampling is always lower than the variance obtained using simple Monte Carlo sampling and proof of this can be found in Ref. 1.

Common Random Number Sampling

Common random number (CRN) sampling, also known as correlated sampling uses two correlated random variables to reduce sampling error. To illustrate the application of CRN,

we start by considering two random variables, X and Y. We also define a third random variable, Z, which is the difference between the two variables.

$$Z = X - Y \tag{11}$$

By considering the means of the random variables we can obtain the following equation.

$$\mu_Z = \mu_X - \mu_Y \tag{12}$$

Our goal is to reduce the sampling error in Z. We note that the variance of Z not only depends on the variance of X and Y, but also on the covariance of X and Y.

$$\mathbb{V}[Z] = \mathbb{V}[X] + \mathbb{V}[Y] - \mathrm{Cov}[X, Y] \tag{13}$$

This implies that if both X and Y are positively correlated, the variance in Z will be *lower* than the sum of variances in X and Y. In CRN, this property is used to reduce the sampling error. To demonstrate its application to variance reduction for a ratio estimation, we consider the following ratio estimator, R, of two one-dimensional functions $f(x)$ and $g(x)$.

$$R = \frac{\langle f(x)\rangle}{\langle g(x)\rangle} = \frac{\sum_i f(x_i)}{\sum_i g(x_i)} \tag{14}$$

Adding ± 1 one to the above expression results in the following equation.

$$R = 1 + \left[\frac{\sum_i f(x_i)}{\sum_i g(x_i)} - 1\right] \tag{15}$$

$$R = 1 + \frac{\sum_i [f(x_i) - g(x_i)]}{\sum_i g(x_i)} \tag{16}$$

By substituting $X = f(x_i)$ and $Y = g(x_i)$, we see that if $f(x)$ and $g(x)$ are positively correlated, then the variance in R can be reduced using CRN sampling.

$$R = 1 + \frac{\sum_i [f(x_i^{\mathrm{CRN}}) - g(x_i^{\mathrm{CRN}})]}{\sum_i g(x_i^{\mathrm{CRN}})} \quad \text{for} \quad \mathrm{Cov}[f, g] \geq 0 \tag{17}$$

Alternatively, if $f(x)$ and $g(x)$ are negatively correlated, we can switch signs to achieve variance reduction.

$$R = -1 + \frac{\sum_i [f(x_i^{\mathrm{CRN}}) + g(x_i^{\mathrm{CRN}})]}{\sum_i g(x_i^{\mathrm{CRN}})} \quad \text{for} \quad \mathrm{Cov}[f, g] < 0 \tag{18}$$

A detailed derivation of CRN and its connection to the use antithetic variables can be found in Ref. 2.

References

(1) Rubinstein, R.; Kroese, D. *Simulation and the Monte Carlo Method*; Wiley Series in Probability and Statistics; Wiley, 2016.

(2) Bratley, P.; Fox, B. L.; Schrage, L. E. *A guide to simulation*; Springer Science and Business Media, 2011.
